# The Impact of the Rs1044457 Polymorphism in the CMPK1 Gene on the Response Rate to Gemcitabine‐Based Chemotherapy in Metastatic NSCLC Patients

**DOI:** 10.1002/ggn2.202400058

**Published:** 2025-04-04

**Authors:** Ghassan Saod Saleh, Fouad Kadhim Gatea, Qasim Sharhan Al‐Mayah, Hayder Lazim

**Affiliations:** ^1^ Ministry of Health, Salahuddin Health Directory Al‐Alam General Hospital Salahuddin Iraq; ^2^ Department of Pharmacology and Therapeutics College of Medicine Al‐Nahrain University Baghdad Iraq; ^3^ Medical Research Unit College of Medicine Al‐Nahrain University Baghdad Iraq; ^4^ Faculty of Health Social Care and Medicine (FHSCM) School of Medicine Edge Hill University Ormskirk L39 4QP UK

**Keywords:** CMPK1 gene polymorphism, gemcitabine response rate, non‐small cell lung cancer, rs1044457 polymorphism

## Abstract

This study aims to evaluate the role of a specific gene polymorphism, Cytidine/Uridine Monophosphate Kinase 1 (CMPK1) rs1044457, in predicting the response to gemcitabine‐based chemotherapy in patients with NSCLC. A total of 98 NSCLC patients are enrolled in the study. Based on their response, patients are classified as either responders or non‐responders. Specific primers are designed to amplify the rs1044457 variant and performed genotyping using restriction fragment length polymorphism (RFLP). The rs1044457 variant showed a statistically significant difference in frequency between responder and non‐responder patients. The mutant genotype (TT) is more frequent in non‐responder patients (18.75%) compared to responder patients (4%), with an odds ratio [OR] of 5.93 (95% confidence interval [CI] = 1.16–30.25, p = 0.032). Additionally, at the allelic level, the mutant allele (T) is more common in non‐responder patients (36.46%) compared to responder patients (23%), with a statistically significant odds ratio of 1.92 (95% CI = 1.03–3.58, p = 0.040). The findings of this study suggest that the mutant allele (allele T) of the rs1044457 variant may serve as a risk factor for resistance to gemcitabine‐based chemotherapy in patients with NSCLC.

## Graphical abstract

Lung cancer is the leading cause of cancer‐related mortality worldwide. Among its types, non‐small cell lung cancer (NSCLC) accounts for the majority of cases. This study focuses on investigating genetic mutations that may influence the response to gemcitabine, a chemotherapy drug commonly used to treat this condition. Specifically, mutations in the rs1044457 variant can categorize patients into two groups: responders and non‐responders.

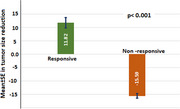

## 1 Introduction

Lung cancer is the leading cause of mortality globally.In particular, non‐small cell lung cancer (NSCLC) accounts for the majority of these cases. While novel drugs targeting specific molecular pathways have become available, cytotoxic chemotherapy remains the primary treatment modality for advanced NSCLC. Historically, gemcitabine and taxane/cisplatin have been conventionally employed in the management of NSCLC, demonstrating comparable treatment efficacy in terms of response rates, patient survival, and time to disease progression, as demonstrated in phase III clinical trials.^[^ ^1^, ^2^ ^]^ Gemcitabine, a pyrimidine analogue (2,2‐difluorodeoxycytidine, dFdC), exerts its cytotoxic effects as an antimetabolite and exhibits activity against a broad spectrum of solid tumours, including pancreatic, oesophageal, breast, and NSCLC.^[^ ^3^ ^]^

Gemcitabine necessitates cellular uptake followed by phosphorylation for its activation. Upon entry into the cell, gemcitabine is phosphorylated by deoxycytidine kinase (dCK) to yield a monophosphate derivative. This monophosphate is subsequently converted to the diphosphate form (dFdCDP) through the action of cytidine monophosphate kinase (CMPK). These phosphorylation steps are critical for the cytotoxic efficacy of gemcitabine. Polymorphisms in the CMPK1 gene, such as rs1044457, can modulate both the activity and expression levels of CMPK1, thereby affecting the therapeutic efficiency of gemcitabine in targeting neoplastic cells.^[^ ^4^, ^5^ ^]^

Based on the information available, the polymorphism of rs1044457 may play a role in the treatment of non‐small cell lung cancer (NSCLC) patients with gemcitabine. Recent findings suggest that dCK polymorphisms, such as the CC genotype of rs1044457, might influence dCK expression and, ultimately, gemcitabine activity in various cancers, including pancreatic cancer.^[^ ^6^ ^]^

In their study, Mirta et al.^[^ ^7^ ^]^ identified an association between the CC genotype of the rs1044457CT 3′UTR SNP and reduced formation and clearance of gemcitabine triphosphate in individuals with various solid tumours. This effect was observed in comparison to individuals possessing the TT genotype who also have cancer.

The presence of the SNP rs1044457 (C>T) in the 3′untranslated region (UTR) suggests that it likely plays a regulatory role in the expression of the CMPK1 gene. The 3′UTR is known to regulate mRNA stability, export to the cytoplasm, sub‐cellular localization, and translation efficiency, thereby influencing the overall synthesis of proteins.^[^ ^8^ ^]^ Consequently, polymorphisms and/or mutations in these regions can have a strong impact on gene expression and the resulting cellular viability, growth, and development

While the specific impact of this polymorphism on gemcitabine treatment in NSCLC patients is not explicitly outlined in the provided search results, the observed associations in other cancer types suggest the potential for similar implications in NSCLC. However, it's important to note that more robust data are needed to confirm the influence of dCK polymorphisms, including rs1044457, on gemcitabine activity in NSCLC patients.^[^ ^9^ ^]^

Genetic polymorphisms play an important role in modulating the activity of transporters and enzymes involved in gemcitabine metabolism, explaining the reported interindividual variability in gemcitabine efficacy. Extensive research has been conducted to investigate the correlation between genetic polymorphisms and the clinical effectiveness of gemcitabine across diverse patient cohorts, including those with lung, pancreatic, and breast cancer.^[^ ^10^, ^11^, ^12^, ^13^, ^14^ ^]^ Notably, studies in pancreatic cancer patients have revealed a link between the CMPK1 gene polymorphism and the survival rate after gemcitabine therapy.^[^ ^15^ ^]^

Therefore, it is reasonable to evaluate that genetic variations in the genes associated with gemcitabine pharmacology may serve as useful predictors for anticipating favourable treatment outcomes with this specific chemotherapeutic agent. The primary objective of the current investigation was to elucidate the impact of the rs1044457 variation on the response to gemcitabine‐based chemotherapy in individuals diagnosed with non‐small cell lung cancer (NSCLC).

## 2 Patients and Methods

### 2.1 Study Design

The present cross‐sectional study was conducted over a period spanning from August 2022 to January 2023, encompassing a cohort of 98 patients of Arabic ethnicity diagnosed with non‐small cell lung cancer (NSCLC) who had undergone four to six cycles of gemcitabine chemotherapy. The study was carried out at multiple clinical sites, including the Oncology Department at Baghdad Medical City, the Oncology Teaching Hospital, the Oncology Department at Al Imamain Al Kadhumain Medical City, and Al Amal Hospital. Furthermore, the molecular assays were conducted at the medical research unit of the College of Medicine, Al‐Nahrain University. The data were then collected in February 2023 and made accessible for research purposes in March 2023.

The inclusion criteria for the study comprised patients with NSCLC who had received four to six cycles of gemcitabine chemotherapy, while those undergoing palliative therapy for advanced NSCLC stages, newly diagnosed patients, and individuals who received gemcitabine therapy for less than four cycles were excluded. The treatment regimen for gemcitabine consisted of an administration of 1000 mg m^−2^ on days 1 and 8, in conjunction with docetaxel at a dosage of 85 mg m^−2^ on day 8, with a three‐week interval between cycles.

Data pertaining to demographic variables such as age, gender, smoking status, comorbidities, family history of malignancy, and occupation, as well as clinical information encompassing treatment protocol, tumour characteristics (including size, site, primary tumour classification, lymph node involvement, and tumour grade and stage), were collected from patient's medical records for analysis.

Nevertheless, substantial inter‐individual variability in patient responses to gemcitabine‐based chemotherapy necessitates the identification of determinants influencing patient outcomes with this agent.

### 2.2 Ethical Considerations and Consent

The samples used in this investigation were acquired specifically for research purposes, and their use was approved by the Ethical Review Board of the College of Medicine at Al‐Nahrain University (IRB/101, No: UNCOMIRB01022024/2nd of August 2022). Before their participation, all patients involved completed informed consent documentation. Furthermore, the authors of this study had no access to any information that could potentially disclose the identities of the participants, neither during nor after the data gathering process.

### 2.3 Patients Categorization

The study participants were classified into two groups based on their treatment response, namely responders and non‐responders. Their treatment responses were assessed in accordance with the response evaluation criteria in solid tumours (RECIST) guidelines by the treating physician. RECIST criteria classified the outcome into Complete Response (CR), Partial Response (PR), Progressive Disease (PD) and Stable Disease (SD).^[^ ^16^ ^]^ However, in our study, responders included SD and PR (there was no CR), while non‐responders included PR, based on the reduction in the primary tumour size.

Tumour response was evaluated through clinical examination and the use of computed tomography (CT) scans at intervals of every two treatment cycles. Additionally, the efficacy and toxicity of the treatment were evaluated, and any chemotherapy‐induced adverse effects were documented.

### 2.4 Blood Samples, Genomic DNA Extraction and Gene Amplification

≈3 mL of venous blood was aseptically collected from each participant and transferred into EDTA tubes to prevent coagulation. Genomic DNA extraction was performed using a commercially available kit (Promega, USA) in accordance with the manufacturer's prescribed protocol. The quantification of the extracted DNA samples was conducted utilizing the QuantiFluor dsDNA System (Promega, USA) and measured on the Quantus Fluorometer (Promega, USA) following the manufacturer's protocols.

The current study used two primers for gene amplification and genotyping of the rs1044457 variant, which was obtained from Macrogen, Korea. The forward primer sequence was (5′‐TGCTCACCAAACGAAGGGTA‐3′) and the reverse primer sequence was (5′‐AGTTCCTTTGAAATTTGGTCCACA‐3). The PCR conditions employed in this study were as follows: an initial denaturation step at 95 °C for 5 min, followed by denaturation at 94 °C for 30 s, annealing at 58 °C for 30 s, extension at 72 °C for 40 s, with the three‐step cycle repeated 30 times, and finally a final extension step at 72 °C for 7 min. Subsequently, the PCR products underwent 2% gel electrophoresis, and the banding pattern was visualized using UV light.

The PCR product was then subjected to digestion by the restriction enzyme. Specifically, 5 µL of the PCR products were combined with 0.1µL of the (*Aci*l, 10U µL^−1^) restriction enzyme obtained from Sibenzyme, Russia. Additionally, 1.5 µL of the 10X restriction buffer and 0.1 µL of bovine serum albumin (BSA) were added to the mixture, which was then brought to a total volume of 15 µL using molecular grade water. Mineral oil (20 µl) was added to all tubes to prevent evaporation. The reaction mixture was incubated in a 60 °C water bath for 3 h. The resulting restriction products were subsequently resolved on a 2% agarose gel.

### 2.5 Statistical Analysis

The statistical analysis was performed using SPSS software version 25.0 (SPSS, Chicago). Continuous data were subjected to the calculation of mean and standard deviation, followed by analysis using the Student's t‐test. The deviation of genotype, namely CC, CT, and TT, in both the responsive and unresponsive groups, from Hardy‐Weinberg Equilibrium (HWE) was assessed using Chi‐square. This test also served for the evaluation of binomial variables expressed as both numerical values and percentages. To explore the association between the CMPK1 rs1044457 and responsiveness to gemcitabine, binary logistic regression was employed to compute the odds ratio (OR) and the corresponding 95% confidence intervals (CI). A p‐value of 0.05 or less was considered indicative of statistical significance.

Continuous data were initially subjected to a normality test using the Shapiro‐Wilk test. Most variables were found to be normally distributed, with the exception of tumour size reduction. Consequently, the comparison between responsive and non‐responsive groups in terms of tumour size reduction will be analysed using the non‐parametric Mann–Whitney U test.

## 3 Results

### 3.1 Demographic Characteristics of the Patients

This study enrolled a cohort comprising 98 individuals diagnosed with NSCLC who underwent treatment involving gemcitabine. The average age of the cohorts of patients exhibiting responsive and non‐responsive reactions to the treatment was found to be 56.04 ± 10.12 years and 57.79 ± 9.02 years, respectively, with no significant difference observed. Furthermore, the distribution of male and female participants was nearly uniform in both groups, displaying no statistically significant divergence. Similarly, the two cohorts demonstrated comparable characteristics in terms of weight, height, and body mass index (BMI), with no statistically significant difference. Additionally, the prevalence of smoking was observed to be common within both cohorts, reported at 72% and 77.07% among responsive and non‐responsive patients, respectively, without significant differences. Moreover, approximately two‐thirds of patients in each group exhibited an Eastern Cooperative Oncology Group (ECOG) score of 2, with no significant differences (**Table** **1**).

**Table 1:** Demographic characteristics of the patients.

Variables		Responsive [n = 50]	Non‐responsive [n = 48]	P‐value
Age (years)	Mean ± SD	56.04 ± 10.12	57.79 ± 9.02	0.369^*^
	Range	38–70	34–70	
Gender	Male	25(50%)	25(52.08%)	0.837^**^
	Female	25(50%)	23(47.92%)	
Weight (kg)	Mean ± SD	66.34 ± 6.49	65.35 ± 7.13	0.476^*^
	Range	54–83	54–82	
Height (cm)	Mean ± SD	162.2 ± 6.03	162.83 ± 5.34	0.584^*^
	Range	150–175	152–180	
BMI (kg/m^2^)	Mean ± SD	25.17 ± 1.44	24.61 ± 1.98	0.110^*^
	Range	21.45–28.65	20.09–29.38	
Smoking	Never	14(28%)	11(22.92%)	0.718^**^
	Ex/current smokers	36(72%)	37(77.07%)	
ECOG	One	9(18%)	4(8.33%)	0.160^**^
	Two	33(66%)	30(62.5%)	
	Three	8(16%)	14(29.17%)	

BMI: body mass index, ECOG: Eastern Cooperative Oncology Group, SD: standard deviation;

* Independent t‐test;

** Chi square test.

### 3.2 Therapeutic and Clinical Characteristics of the Patients

The prevalence of hypertension and ischemic heart diseases (IHD) exhibited a slightly higher incidence among non‐responsive patients (37.5% and 14.58%, respectively) compared to responsive patients (24% and 10%, respectively); however, these disparities did not reach statistical significance. Conversely, other comorbidities were non‐significantly more prevalent among responsive patients than non‐responsive patients (8% vs 6.35%). The initial tumour size in the responsive group was (45.15 ± 36.67 cm), comparable to that of non‐responsive patients at (42.26 ± 33.18 cm), with no statistically significant variance. Following 4–6 cycles of treatment, the mean final tumour size in responsive patients reduced to (34.6 ± 30.94 cm), significantly lower than that of non‐responsive patients (57.86 ± 43.8 cm) with a highly significant difference. Approximately three‐fourths of the patients in either group received 6 treatment cycles, with no significant discrepancy between the two groups (**Table** **2**).

**Table 2:** Clinical characteristics of the patients.

Variables		Responsive [n = 50]	Non‐responsive [n = 48]	P‐value
Comorbidities	No comorbidity	12(24%)	10(20.83%)	0.821^**^
	Diabetes mellitus	12(24%)	18(37.5%)	0.147
	Hypertension	15(30%)	15(31.25%)	0.893
	Ischemic heart disease	5(10%)	7(14.58%)	0.489
	Others	4(8%)	3(6.35%)	0.737
Initial tumor size, cm	Mean ± SD	45.15 ± 36.67	42.26 ± 33.18	0.151^*^
	Range	3.48–120.96	14.94–127.6	
Final tumor size, cm	Mean ± SD	34.6 ± 30.94	57.86 ± 43.8	**0.003** ^*^
	Range	0.5–101.92	16.4–142.74	
Treatment cycles	4	13(26.67%)	9(13.33%)	0.390^**^
	6	37(73.33%)	39(86.67%)	

SD: standard deviation;

* Independent t‐test;

** Chi square test.

### 3.3 Reduction in Tumour Size

**Figure 1:**
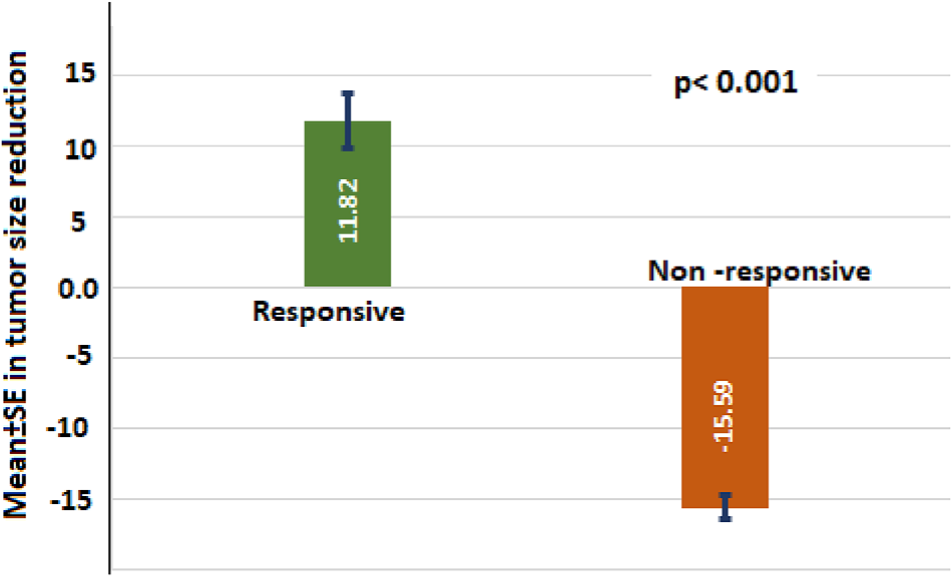
Reduction in tumour sizes in responsive and non‐responsive patients.

The study observed a discernible reduction in tumour size as determined through CT scan analysis, with the responsive group exhibiting an average reduction of 11.82 ± 13.98 cm (ranging from 2.27 to 57.51 cm), as opposed to the non‐responsive group, which demonstrated a reduction of −15.31 ± 6.27 cm (ranging from −2.7 to −24.18 cm). The application of the Mann–Whitney U test yielded a notably significant difference in tumour size reduction between the two groups (**Figure** **1**).

### 3.4 Correlation of Tumour Reduction with Other Variables

**Figure 2:**
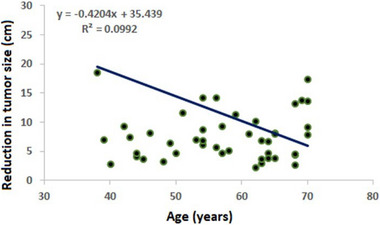
Scatter plot and regression line between age and tumour size reduction in responsive patients.

**Figure 3:**
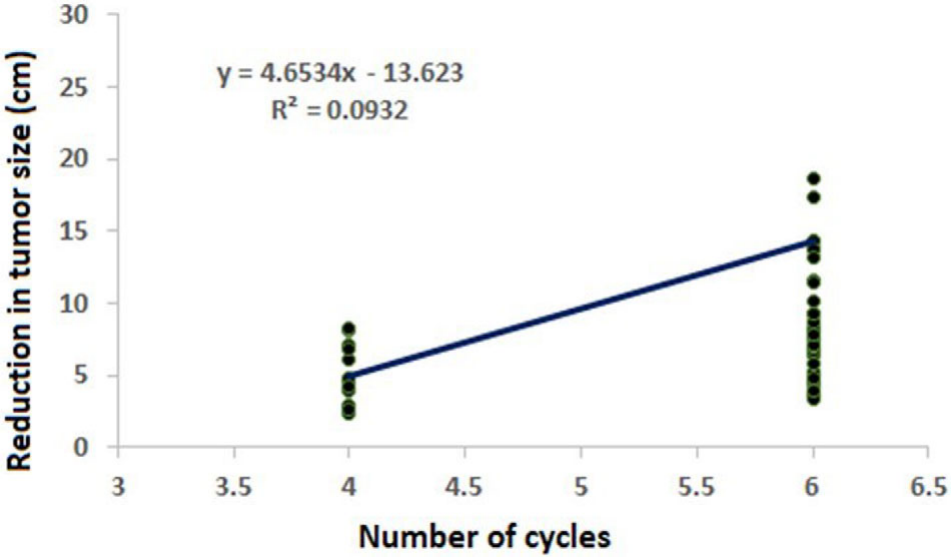
Scatter plot and regression line between number of cycles and tumour size reduction in responsive patients.

Pearson's correlation test was used to investigate the possible correlation between tumour reduction and various continuous variables. Among responsive patients, the analysis revealed a negative correlation between tumour size reduction and age (r = −0.315, p = 0.026), as well as a significant positive correlation with treatment cycles (r = 0.305, p = 0.031). Conversely, in non‐responsive patients, tumour size reduction exhibited no significant correlation with any of the included variables as shown in **Table** **3**, **Figure** **2**, and **3**.

**Table 3:** Association between tumour size reduction and other continuous variables in responsive and non‐responsive patients.

Variable	Responsive		Non‐Responsive	
	Coefficient	p‐value	Coefficient	p‐value
Age	**−0.315**	**0.026**	−0.159	0.279
Weight	−0.031	0.830	0.181	0.218
Height	−0.177	0.218	−0.021	0.889
BMI	0.181	0.209	0.219	0.135
No. of cycles	**0.305**	**0.031**	0.227	0.126
ECOG score	−0.221	0.118	−0.256	0.080

Pearson's correlation test.

### 3.5 Association of Tumour Reduction with Gender, Smoking and Comorbidity

In the context of responsive and non‐responsive groups, the findings revealed notable distinctions in tumour size reduction based on gender, smoking history, and comorbidities among the patients. Specifically, females exhibited a greater reduction in tumour size compared to males in the responsive group, displaying mean values of 15.53 ± 16.26 cm and 8.22 ± 8.97 cm, respectively, which was statistically significant. Furthermore, non‐smoker patients demonstrated a significantly higher level of tumour size reduction in comparison to ex/current smokers in both responsive and non‐responsive patients. Additionally, patients without comorbidities exhibited a higher mean reduction in tumour size (12.4 ± 13.01 cm) than those with comorbidities (8.04 ± 4.55 cm), although the difference was not significant (**Table** **4**).

**Table 4:** Association of tumour reduction with gender, smoking and comorbidity.

Variables		Responsive	Non‐responsive
Gender	Males Females	8.22 ± 8.97 15.53 ± 16.26	−15.65 ± 5.54 −14.95 ± 7.09
	p‐value	0.055	0.705
Smoking	Yes No	7.67 ± 4.1 13.51 ± 15.47	−17.34 ± 35.79 −13.11 ± 6.14
	p‐ value	**0.026**	**0.018**
Comorbidity	Yes No	8.04 ± 4.55 12.4 ± 13.01	−14.93 ± 6.71 −15.96 ± 5.6
	p‐value	0.157	0.586

Non‐parametric Mann–Whitney test was used for comparison.

### 3.6 CMPK1 Gene and rs1044457 Polymorphism

**Figure 4:**
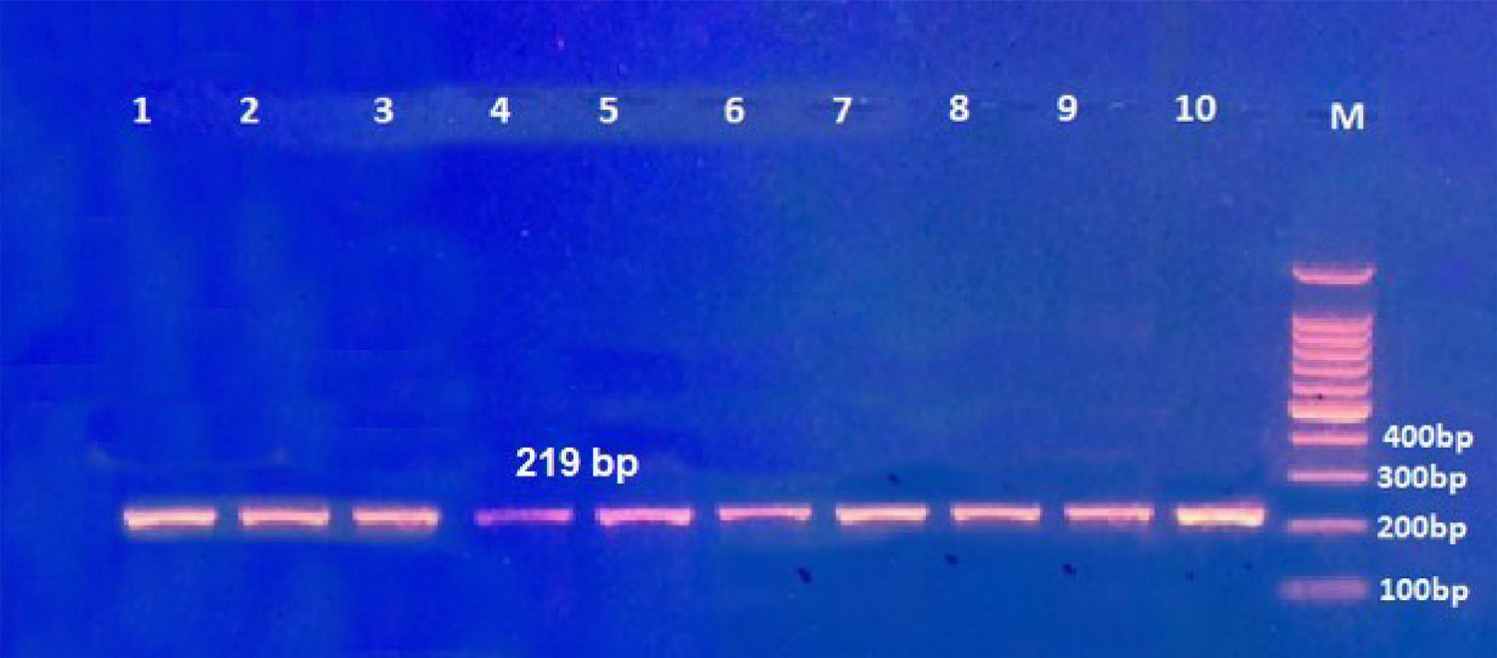
Gel electrophoresis of rs1044457 gene plymorphism amplified with a specific pair of primers using conventional PCR. The PCR product was stained with ethidium bromide. The fragment length was 219 bp. M: 100 bp DNA ladder. Samples were randomly selected for responsive (lanes 1–5) and non‐responsive patients (lanes 6–10).

**Figure 5:**
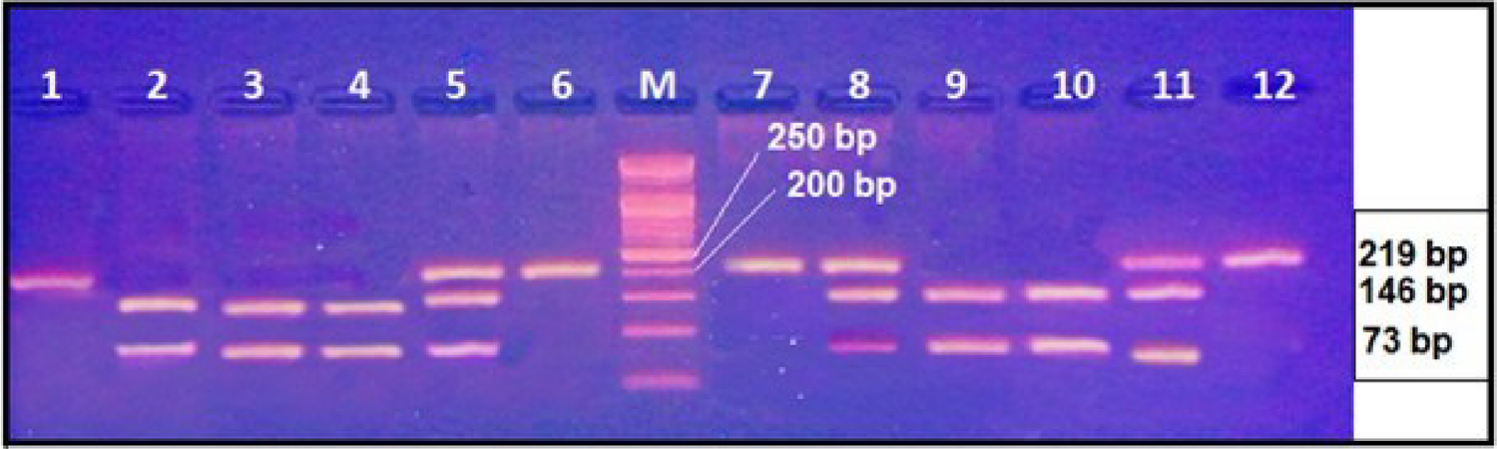
Genotyping of rs1044457 gene polymorphism after digestion with AciI restriction enzyme and stained with ethidium bromide. Lanes 1, 6,7 and 12: TT genotype; lanes 2,3,4,9and 10: CC genotypes, lanes 5,8 and 11: CT genotype; M: 50 bp DNA ladder. The fragment length was 219 bp. M: 100 bp DNA ladder. Samples were randomly selected for responsive (lanes 1–6) and non‐responsive patients (lanes 7‐12).

Two primers were employed for the amplification of this polymorphism through conventional PCR. Subsequent gel electrophoresis of the PCR product demonstrated a fragment length of 219 base pairs (bp), as shown in **Figure** **4**. Genotyping was executed utilizing the Restriction Fragment Length Polymorphism (RFLP) technique. Subsequent digestion with the *Aci*l restriction enzyme revealed the presence of three genotypes: CC, CT, and TT, as illustrated in **Figure** **5**.

The prevalence of the wild type of CC genotype was observed to be higher in responsive patients compared to non‐responsive patients, accounting for 58% and 45.83% respectively; however, this disparity did not yield statistical significance. Conversely, the occurrence of the mutant genotype (TT) was notably greater in non‐responsive patients (18.75%) in comparison to responsive patients (4%), exhibiting a statistically significant difference (OR = 5.93, 95% CI = 1.16–30.25, p = 0.032). This polymorphism appears to demonstrate dominant inheritance, as evidenced by the higher prevalence of the CC+CT genotype in responsive patients (96%) versus non‐responsive patients (84.62%), with a significant distinction (OR = 5.53, 95%CI = 1.13–27.14, p = 0.035). Furthermore, at the allelic level, the mutant allele (T) was notably more prevalent in non‐responsive patients (36.46%) than in responsive patients (23%), with a statistically significant contrast (OR = 1.92, 95%CI = 1.03–3.58, p = 0.040), as shown in **Table** **5**.

**Table 5:** The frequency of different genotypes and alleles of the polymorphism rs1044457 in responsive and non‐responsive patients.

rs1044457		Responsive [50]	Non‐responsive [48]	*P*‐value	OR [95%CI]
Genotypes	CC CT TT	2**9**(58%) 19 (38%) **2**(4%)	22(45.83%) 17(35.42%) 9(18.75%)	0.100 0.706 **0.032**	1.0 1.18 (0.50–2.78) 5.93 (1.16–30.25)
Dominant model	CC+CT TT	48(96%) **2**(4%)	39(84.62%) 9(18.75%)	**0.035**	1.0 5.53 (1.13–27.14)
Recessive model	CC CT+TT	29(58%) 21(42%)	22(45.83%) 26(54.17%)	0.229	1.0 1.63 (0.73–3.63)
Alleles	C T	77(77%) 23(23%)	61(63.54%) 35(36.46%)	**0.040**	1.0 1.92 (1.03–3.58)

Binary logistic regression test.

All responders exhibited some degree of tumour size reduction, whereas all non‐responders experienced tumour enlargement. Based on these observations, we categorized the patients according to their tumour size outcomes and analysed the correlation between these outcomes and the different genotypes and alleles of the rs1044457 polymorphism.

The distribution of genotypes was assessed in both responsive and non‐responsive groups. To calculate the Hardy–Weinberg equilibrium (HWE) the chi‐square test statistic was calculated to be 5.488 with 2 degrees of freedom (d.f.), resulting in a *p*‐value of 0.064. This *p*‐value indicates that the observed differences are not statistically significant at the conventional significance level of 0.05. Therefore, it can be concluded that there are no significant differences between the Hardy–Weinberg equilibrium (HWE) and the dataset under consideration. Consequently, our dataset complies with the Hardy–Weinberg equilibrium (HWE) based on the results of the statistical analysis.

## 4 Discussion

Gemcitabine is an important component in the treatment of advanced NSCLC. A meta‐analysis showed that gemcitabine‐based chemotherapy resulted in a median survival of 9 months, compared to 8.2 months with non‐gemcitabine combinations in advanced NSCLC patients.^[^ ^17^ ^]^ This highlights the potential benefits of gemcitabine‐based regimens in NSCLC treatment.

The impact of genotypes present at the rs1044457 locus on drug pharmacokinetics has been emphasized in various studies.^[^ ^7^, ^8^ ^]^ Although there is evidence suggesting that CMPK1 polymorphisms affect chemotherapy outcomes, specific research focusing on the rs1044457 polymorphism in the context of metastatic non‐small cell lung cancer (NSCLC) is not directly available in the current literature.

This study identified three distinct polymorphisms CC, CT, and TT through genotype analysis. Here, CC represents the wild‐type genotype, while TT denotes the mutant form. These polymorphisms are observed in both the responsive and unresponsive groups. Furthermore, the distribution of these genotypes within each group adheres to the principles of Hardy–Weinberg equilibrium.

The most interesting finding in the current study was that the mutant genotype (TT) of CMPK1 rs1044457 gene polymorphism had a higher prevalence in non‐responsive patients (18.75%) compared to responsive patients (4%). This difference was found to be statistically significant (odds ratio = 5.93, 95% confidence interval = 1.16–30.25, p‐value = 0.032). However, there was no direct comparison between TT‐bearing non‐responsive and TT‐bearing responsive, but the categorization of groups into responsive and non‐responsive was almost entirely based on the tumour size reduction. Thus, there was an indirect comparison.

A study focusing on advanced NSCLC patients treated with gemcitabine or taxane/cisplatinum found that polymorphisms in CMPK1 were associated with differential survival outcomes. Specifically, the wild‐type genotypes of CMPK1 IVS1+1057 and IVS1‐928 were linked to shorter overall survival in patients treated with gemcitabine/cisplatinum, but not in those treated with taxane/cisplatinum. This indicates that CMPK1 polymorphisms may have a specific impact on gemcitabine‐based treatments in NSCLC patients.^[^ ^18^ ^]^

A recent study investigated the role of CMPK1 polymorphisms in gemcitabine‐based chemotherapy for HER2‐negative metastatic breast cancer patients. The research found that the rs1044457 polymorphism in CMPK1 was strongly associated with progression‐free survival (PFS). Patients with the CC genotype showed a higher 6‐month PFS rate (78.9%) compared to those with CT and TT genotypes (55.9%). This suggests that the rs1044457 polymorphism may influence the response to gemcitabine‐based treatments, although in breast cancer rather than NSCLC.^[^ ^4^ ^]^

CMPK1 plays a key role in the phosphorylation of nucleoside analogues like gemcitabine. Specifically, it converts gemcitabine monophosphate (dFdCMP) into gemcitabine diphosphate (dFdCDP), a critical step in the drug's activation. Polymorphisms in the CMPK1 gene, such as rs1044457, may potentially alter the enzyme's activity, expression, or stability. In patients carrying the T allele of this polymorphism, the conversion of gemcitabine monophosphate (inactive) to gemcitabine diphosphate (active) might be less efficient. This could result in lower intracellular levels of the active drug, potentially reducing its cytotoxic effects and leading to poorer therapeutic outcomes. Furthermore, this polymorphism may also affect the transcriptional regulation of CMPK1, resulting in lower expression levels of the enzyme. This, in turn, could similarly reduce gemcitabine activation and compromise its efficacy.

According to the results of the present study, age was negatively associated with a reduction in tumour size while there was a positive correlation between the reduction in tumour size and the number of treatment cycles. Our findings agreed with other studies performed on elderly patients with various malignancies showing that elderly patients benefit from chemotherapy to a similar extent as younger patients, with manageable side‐ effects.^[^ ^19^, ^20^ ^]^

The vast majority of these subgroup analyses demonstrated that the efficacy results are similar in patients aged <70 years old and in those aged ≥70 years.^[^ ^21^, ^22^ ^]^ In these trials, no difference in survival was observed according to age. The only study showing a shorter survival in elderly patients is the combined analysis of two Southwest Oncology Group trials. One trial compared cisplatin alone to cisplatin‐vinorelbine, and the other compared cisplatin‐vinorelbine to carboplatin‐paclitaxel.^[^ ^23^ ^]^ A study conducted in China showed that age (<70 vs ≥70 years) did not affect treatment outcomes, but older adults were more likely to experience adverse events, highlighting the importance of patient selection to inform treatment decisions.^[^ ^24^ ^]^

In the present study, a notable difference in tumour reduction was observed between females and males, favouring males, although the *p*‐value exceeded the threshold for statistical significance. Previous studies have shown that females tend to exhibit slower drug clearance for gemcitabine, resulting in higher systemic drug exposure and increased toxicity. In contrast, males typically metabolize and eliminate gemcitabine more efficiently, which may enhance its therapeutic effects while reducing side effects.^[^ ^25^ ^]^ Additionally, females with NSCLC are more likely to have EGFR mutations, which respond better to targeted EGFR inhibitors rather than chemotherapy. On the other hand, males with NSCLC more frequently exhibit KRAS mutations or smoking‐related carcinogenesis, making them more reliant on chemotherapy than on targeted treatments.^[^ ^26^ ^]^

The recommended number of treatment cycles for gemcitabine therapy in NSCLC ranges from four to six. The longer the duration of treatment, the greater the response. Chemotherapy selectively targets cells undergoing mitosis, the process of cell division. Resting cells are not susceptible to destruction. Therefore, with each successive cycle, a greater number of tumour cells are eradicated. However, with increased doses of gemcitabine, pulmonary toxicities have been reported such as bronchospasm, capillary leak syndrome, and non‐cardiogenic pulmonary edema.^[^ ^27^ ^]^

Alternatively, a study conducted by Woo et al.^[^ ^14^ ^]^ found that individuals with the CC genotype, diagnosed with pancreatic cancer and undergoing treatment with a combination of gemcitabine and erlotinib or fluoropyrimidines, experience improved overall survival rates and a longer time to tumour and disease progression compared to individuals with the TT genotype.

Taken together, these findings highlight the significance of the CMPK1 rs1044457 gene polymorphism in response to gemcitabine‐based chemotherapy. It is advisable to consider alternative chemotherapy options for NSCLC patients who carry the T allele of this polymorphism. The role of the rs1044457 polymorphism in gemcitabine treatment of NSCLC patients is an area of ongoing research, and while initial findings are promising, further studies are required to establish its specific impact in this context.

### 4.1 Limitations of the Study

The study's time constraints prevented an analysis of the relationship between the rs1044457 polymorphism in the CMPK1 gene and overall survival. Additionally, the relatively small cohort of 98 patients may have limited the study's statistical power and its generalizability to the broader NSCLC population. This small sample size also restricted the ability to perform multivariate analyses and adjust for potential confounding factors, such as comorbidities, lifestyle factors (e.g., smoking), and other genetic variations.

When considering genetic polymorphisms, such as rs1044457, in treatment responses, it's essential to account for the variability among different ethnic groups and subpopulations. This variability underscores the importance of personalized medicine tailored to genetic diversity.

## Conflict of Interest

The authors declare no conflict of interest.

## Author Contributions

G.S.S was responsible for the conceptualization, data curation, formal analysis, investigation, methodology, validation, visualization, and writing of the original draft, as well as reviewing and editing the manuscript. F.K.G contributed to the conceptualization, data curation, formal analysis, resources, supervision, validation, visualization, and writing of the original draft, along with reviewing and editing the manuscript. Q.S.A was involved in the conceptualization, data curation, formal analysis, methodology, resources, supervision, validation, visualization, and writing of the original draft, and also participated in the review and editing process. H.L took part in the conceptualization, data curation, formal analysis, methodology, project administration, software development, validation, visualization, and writing of the original draft, while also reviewing and editing the manuscript.

## Supporting information



Supporting Information

## Data Availability

The data that support the findings of this study are available in the supplementary material of this article.
